# Revision of Conception of Gradual Formation of Actions for Education and Psychological Development

**DOI:** 10.3389/fpsyg.2020.01887

**Published:** 2020-07-30

**Authors:** Yulia Solovieva, Luis Quintanar

**Affiliations:** Faculty of Sciences for Human Development, Autonomous University of Tlaxcala, Tlaxcala, Mexico

**Keywords:** formation of action, orientation, activity theory, psychological development, education

## Abstract

The psychological conception of the formation of action by stages has been one of the most significant contributions to activity theory. This conception can be understood in two ways: (1) in a broad way as general psychological conception and (2) in a straight way as a conception of the process of teaching and learning according to activity theory. We propose to consider the union of these two conceptual possibilities as a general methodological proposal for the study of development. The article offers a revision of the conception of gradual formation of action by stages according to modern educational needs in the sense of a union between Vigotsky’s and Galperin’s conception of psychological development. The article revises the possibility for usage of this methodology in a broad way as a general psychological conception, which might include modes of positive development together with developmental difficulties. From the point of view of an activity theory approach, brain functional systems might be understood as psycho-physiological dynamic mechanisms of actions and operations fulfilled by a subject. At the same time, the subject’s own action is always accomplished within the context of one or another cultural activity. The conception of the gradual formation of action by stages helps to plan and organize specific types of interactions between child and adult in significant cultural situations. The stages of formation of cultural action, discussed in the article, are: material action with objects, materialized actions with external symbols, perceptive concrete action with concrete images, perceptive symbolic action with perceptive symbols, and verbal actions. The orientation base of action is an essential part of action on each level. These stages differ from the original proposal within Galperin’s theory and offer a possibility to work with different kinds of actions: practical, intellectual, artistic, and physical actions. These types of actions might be used in educational processes in optimal situations and in situations with children with developmental difficulties. Our proposal opens a question about the types of actions which might be considered during the formation process, such as practical and intellectual actions. We discuss the usefulness of this psychological conception for the methods of assessment, correction, and teaching, which contribute to the development of the child.

## Introduction

The conception of the gradual formation of mental action by stages is one of the most known conceptions within the general theory of psychological activity. This conception was proposed and studied by Piotr Galperin. It’s important to revise, firstly, the relation between activity theory and cultural historical psychology, and afterward to discuss theoretical and practical implications of this conception. Finally, it’s important to stress that few psychological modern studies applied to education use this conception as a real concrete methodology related to the cultural-historical paradigm in psychology.

From the point of view of historical-cultural psychology, the principal object of psychological study is the process of psychological development. It’s possible to affirm that development is both the object of psychological study and the method of research. Specialists of historical-cultural psychology were always interested in the research of development of such a phenomenon when the final process and final results aren’t yet known. From the point of view of cultural-historical psychology, the process of psychological development is, first of all, a cultural process, in which the child acquires the ideal possibility of mediatized regulation of self-individual activity.

According to Vigotsky (1996), the process of psychological development passes through inter-psychological and extra-psychological stages. During these stages, the child is regulated by external cultural means. The inter-psychological stage is the stage of regulation of a child’s activity by an adult’s external language, and the extra-psychological stage is the stage of regulation of a child’s own external means. The most important means for such inter- and extra-regulation is language. As an adult uses the language for regulation of a child in the inter-psychological stage, the child uses their own external egocentric language for self-regulation at the extra-psychological stage. Later on, the child might be able to regulate self-activity using internal language (inner speech), which represents the stage of intra-psychological development. So, Vigotsky (1996) argued that each psychological process appears on the scene of development twice: firstly, as external, material, and social processes, and later on, as internal, individual, and ideal processes.

In general, the changes from the inter-psychological up to the intra-psychological stage of development represents the conception of interiorization as the line of potential cultural development. External psychological processes at the beginning converts into internal psychological processes at the end. This conception, for many psychologists, seems to be the basis of the process of cultural development, as the child acquires human culture from external factors and incorporates these into their internal plan of their own psychic life. The problem with this position is that human culture includes not only language and verbal communication, but also different kinds of actions, such as, for example, intellectual, artistic, practical, and communicative actions. It’s possible to assume that language might exist on an internal plane, but what happens to the other kinds of actions, such as intellectual and artistic actions? Can they be fulfilled in the inner plane? If we say so, we may fall into a methodological mistake, as we normally see “material external” processes and results of practical and intellectual actions of the child. How do these processes pass into the internal plane? Or do they not pass the internal plane at all? If so, we have to choose between two options: (1) acknowledge that Vigotsk’y conception of interiorization is wrong or (2) accept modifications and corrections of this conception.

The authors of this article prefer to accept the second option and affirm that Vigotsky was partly right and that his conception must be completed and modified. Galperin’s proposal for the gradual formation of mental actions by stages is one of the attempts to solve the problem of modifications and complements for Vigotsky’s conception of interiorization.

In our opinion, activity theory is the optimal paradigm in modern psychology, which not only continues but also strengthens Vigotsky’s conception of psychological development. Activity was the new theoretical concept in replacement of the concept of isolated functions, both inferior and superior. Activity became a radical form of interpretation of the nature, structure, and functioning of psychological processes. Activity became the paradigm for study as the object of general psychology (Leontiev, 1984). Activity became the methodological scheme for research in applied fields of psychology, such as educational and developmental psychology (Leontiev, 2010). Psychological development or cultural development, according to Vigotsky, can take place only within different kinds of cultural activities, in which the child takes part during their ontogenetic development. Not verbal communication by itself and as itself, but specific cultural activity directed to a goal and accompanied by non-verbal and verbal communicative means would lead to further psychological development. Introduction and formation of new actions would be a fundamental task of psychological development and education (Talizina, 2018).

As in the case of psychological development, the conception of the gradual formation of mental action might represent an object of psychological study and, at the same time, the method of organization for research. Such research might be conducted starting with the absence of mental action or total absence of psychological action. This kind of research might be conducted in cases of children exhibiting normal development and children with difficulties in psychological developmental and school learning (Solovieva and Quintanar, 2018, 2019a). It would also be possible in adults in cases of the formation of new psychological actions and in adults experiencing disintegration of the process of fulfilment of different actions due to pathological stages of the central and peripheral nervous system, in other words, in cases of neuropsychological rehabilitation of adult patients (Tsvetkova, 1977; Leontiev and Zaporoztets, 2016; Luria, 2016). For example, this might happen in cases of educational psychology, when the formation of action of classification might be studied (Solovieva, 2014). In developmental psychology, the actions of play might be introduced and studied by stages. Examples of the introduction of graphic action (Solovieva and Quintanar, 2019b) and play actions were previously described (Solovieva and Quintanar, 2017, 2019c).

The goal of this article is to contribute to the argument for usage of Galperin’s conception of the gradual formation of mental actions by stages for the field of education and developmental psychology. The authors express original ideas about the actualization of understanding of the stages of formation of mental actions and provide some examples from their own studies with children of pre-school and school ages.

## Conception of Interiorization of Actions

The conception of interiorization is recognized as the general line of cultural development according to Vigotsky, which helps to define the central role of verbal communication in psychological development. This happens because language is only psychological process which achieves complete interiorization at the level of internal language. At this stage, internal language plays the role of mediatization and regulation of personality (Vigotsky, 1993). In his description of the process of inner speech, Vigotsky has brilliantly shown examples of functioning of internal language in a human’s life and intrapersonal communication in adults with cases of possible external manifestations of internal language as a necessity of extra regulation by external means. He has also shown specific features of internal language as agglutination, predominance of personal sense, abbreviation, reduction of grammar, affective nature, and relation to the sphere of motives of personality. However, at that time there was no experimental proof of this hypothesis about the development of external language into internal in specific cases of children or adults. All Vygotsky’s ideas were exceptional ideas without experimental demonstration of such gradual changes in real subjects, who would pass through different stages of external and internal language.

At the same time, the whole spirit of Vigotsky paradigm of historical and cultural development required experimental demonstration and modification. Since psychological development became an object and the method at the same time, it was necessary to provide and carry out studies, which would show the “technique” and the possibilities of guided psychological development under the conditions of psychological experiments. Galperin’s conception provides a consistent platform for the guided introduction and formation of new actions in different periods of development and different situations of human life. At the same time, if an action already exists in a child’s experience, Galperin’s conception shows how to achieve further development of the action with characteristics of high automatization, critic, consciousness, and rationality. These features were termed secondary features of the developed action (Talizina, 1984; Galperin, 1998). Precise characteristics of the actions depend on the content of each intellectual or practice action and on the context of the social situation of the usage of the action.

It’s important to remember that it was Galperin who formulated the hypothesis of the function of control as the function of attention. Galperin expressed that external forms of control of a child’s action might be interiorized into internal forms of control, when the child may fulfill an action with no need for external means of control. The child might acquire an internal plan of control and this form of control might explain the functions of the process of attention. Galperin has experimentally demonstrated the stages of formation of the action of control from materialized into internal mental control. Galperin stressed that only internal control might be referred to as attention (Galperin and Kabilnitskaya, 1974).

According to Vigotsky’s conception, language played a central role in ontogenetic development and development of consciousness (Vigotsky, 1991). If we remember the relation between Galperin’s conception of the gradual formation of mental actions by stages, we might notice that the stage of verbal action was not the first stage of such formation, but appeared as the result of different changes and the transformation of material and perceptive actions. In this conception, verbal actions appear only after the formation of material and perceptive actions. This position contradicts Vigotsky’s position about the role of language in psychological development. How is it possible to relate this conception with Vigotsky’s opinion on the central role of language for ontogenetic development?

According to psychological data, there is a strong evidence to underline that language acquisition is not the only crucial stage in ontogenetic development. Verbal development is very important for psychological development, but it’s possible to affirm that it’s the only line of such development. For example, verbal communication is only one of many possible ways to communicate culturally. Communication is possible without language in any circumstance and at any age (Tomasello, 2013). Non-verbal communication is an initial and basic form of communication in ontogenetic development (Lisina, 2009). The first kind of psychological activity was determined as the activity of close emotional non-verbal communication between adult and child, which should emerge during the first months of a child’s life as the fundamental activity to guarantee the psychological development of activity and personality during the first year.

Lisina (2009) has shown that verbal development doesn’t take place as an auto-sufficient predominant line of development, but inside the content of the first kind of cultural activity of a child, as an activity of joint cultural communication. The forms of communicative activity change according to ontogenetic periods of development (Lisina, 2013). From the very beginning, the presence of goals of communication with an adult offers the basis for the later appearance of verbal expressions of the child. The absence or insufficient levels of goals for non-verbal personal communication contributes to developmental delay or problems with language acquisition.

The works of Lisina (2009, 2013) serve as a positive example of the possibility of studying different types of psychological activities during different periods of ontogenetic development. The central topic of this article is related to one such period, that is, the period of directed school learning. During this period, the child starts to encounter specific kinds of human experiences using conceptual knowledge (Davidov, 2000).

According to activity theory applied to the teaching and learning process, school learning is the period of guided activity of school learning, when the children form intellectual reflexive actions with scientific concepts (Davidov, 2008; Talizina, 2019). Galperin’s conception of the gradual formation of mental actions is closely related to this period of the child’s development, even though this conception might be applied to other periods and conditions of ontogenetic development.

Let us revise the conception of the formation of mental actions, proposed by Galperin, in detail.

First of all, we can remember the idea of the invariant psychological structure of psychological action, which includes motive, objective, orientation, and result (Leontiev, 1984). The levels of realization of action are the level of action by itself characterized by the presence of a conscious goal, level of operations characterized by conditions and means of fulfilment of the action, and level of psychophysiological mechanisms as the level of a functional system of diverse physiological and functional brain mechanisms of the action (Leontiev, 1984). Functional parts of the action using the process of realization of each psychological action is represented in part by orientation, execution, and verification with possible control (Talizina, 2019).

Orientation, as the structural component of an action, has its own features in the case of intellectual actions. In these actions, on the stage of initial formation or introduction, there is a need to separate and present it as an independent action or even as a system of specific directed actions with their own specific goals. For example, in the case of intellectual action of classification, it’s necessary to confirm the action of identification of essential features for classification. The child has to learn to identify objective features in the series of objects in order to decide the conceptual class for the inclusion of each object. Traditional teaching does not pay attention to the action of identification of essential features and rejection of irrelevant features before passing to the action of classification by itself.

According to Galperin’s proposal, orientation might be external or internal (already acquired) by the pupil. The plans of external orientation are materialized and verbal. The materialized plan includes three different forms of the action: material, materialized, and perceptive or written. The verbal plan might be orally represented in loud speech or silence speech. While gradual interiorization should be guaranteed for the whole structure of the action, such as motive, objective, and result, it is not always necessary.

In many cases, the other structural elements of actions (motive, objective, and result) are represented by external material, materialized, perceptive, or verbal means. Such means might be instruments, words, images, symbols, objects, and so on. Such external objects or external results of actions would never pass into an internal ideal plan. Only the orientation of action, as in speaking about practical or artistic actions, might be interiorized as an ideal internal process of the subject as the result of the interiorization of the action. It’s important to realize the differentiation of actions which may and may not form through different stages of formation as the process of interiorization.

There is only one kind of psychological action which might completely pass to the level of ideal actions. Such actions are intellectual actions with concepts. We believe that, regarding Galperin’s conception of gradual formation of mental actions, it’s necessary to stress that the process of gradual formation refers specifically to intellectual actions and not to all kinds of actions. Not all actions pass through all stages described by Galperin and achieve the level of mental ideal actions, instead remaining as only intellectual actions. In the case of practical and artistic actions, it would be possible to speak of automatization instead of interiorization. Only the part of orientation, as the central part of the action, would possibly achieve the internal ideal level or the level of image of the action (Galperin, 2000).

This previous consideration allows us to assume two important comments, which would help to better understand Galperin’s idea.

The first comment is that the gradual formation with all described stages refers only and specifically to intellectual actions. It’s obvious that practical and artistic actions might also be formed by stages, but the stages would be different from the stages presented in the literature for intellectual actions. In each concrete case, it’s necessary to revise and define the stages for the formation of practical or artistic actions.

The second comment refers to the stages described by Galperin in his conception. It would be much better to refer to them as conceptions of the gradual formation of orientation by stages instead of the gradual formation of actions. Orientation is a general aspect of each psychological action and the only one which might be consistently accomplished at an ideal internal level on the late stage of its formation.

The text part of the article is dedicated to the consideration of the stages of formation of action and orientation as the central component of the action.

## The Content of the Form of Psychological Action

Galperin (2000) proposed the differentiation of the form of fulfilment of the action and the stages of formation of the action. Between the forms of realization of the action we find four basic forms: (1) material, materialized, perceptive, or written action; (2) oral verbal action; (3) silent verbal action; and (4) internal mental or internal perceptive action. This last form of action is also determined by Galperin as an ideal action.

Table 1 presents the features and examples of these actions. We find it useful to offer detailed explanations of the terms “form” of action and “plan” of action. The content of this table will help the reader to distinguish these terms, proposed by Galperin in his works. When someone uses the term “materialization” or “exteriorization” of the action, that means that it’s necessary to pass from an “internal” to “external” form of action, that is, pass form the level of oral or silent speech to the level of material, materialized, perceptive, or written action. Same a transformation might also be achieved while transforming mental (ideal) action into the form of external action: material, materialized, perceptive, or written. These kinds of transformation are very useful during the process of formation of action by stages.

**TABLE 1:** Forms of realization of psychological action.

**Form of action**	**Variant of form (plan of action)**	**Example of action**
External	Material	Play with toys
External/ideal	Perceptive	Note differences in pictures
External	Materialized	Playing chess
External	Written language	Write down conditions of mathematical task
Verbal	Verbal oral	Answer questions about the content of the text
Verbal	Verbal silent	Count sentences in a known song
Internal/ideal	Internal image	Imagine fantastic animal
Internal/ideal	Internal verbal concept	Find a solution to a theoretic problem

The forms of psychological action, as shown in Table 1, should be understood as the forms of existence of any psychological action. The understanding of the forms of psychological action helps to understand dialectic logic of the process of formation of action by stages. It’s also possible to discuss these principal forms of existence of each intellectual action as external actions (material, materialized, perceptive, or written): oral action, silent verbal action, and ideal action. We believe that this is a rather complicated part of Gaplerin’s theory. These variants of forms might also be called “plans” of action. Often, both terms are used in psychological studies and literature without any distinction.

It is necessary to accept that the action can exist in one of these forms and there two forms of this existence: as an independent previously confirmed action and as an action guided from an external form into an internal form. According to Galperin, it is possible to differentiate the terms “ideal” and “internal.” For example, a perceptive action is ideal as it occurs with images, but this action isn’t an internal action if a perceptive image is present in front of the subject (Table 1). An example of this situation is when the child is asked to compare two pictures of elephants and the child really sees the pictures. This is an ideal perceptive action with the image of elephant. On the contrary, when the child is asked to imagine an elephant without any picture present, he or she would use an internal plan of the action or the plan of ideal internal image. In Table 1, an example is given about the imagining of a fantastic animal.

We find it interesting and important to comment on the most complex form of psychological action, the level of internal ideal action. Galperin (2000) proposes two different variants of this form: ideal action with internal image and ideal action with internal concept. We understand internal action with ideal image as an action of imagination or as creative work with an image. This would include the planning of a picture by an artist or the internal structuring of a stage in a dramatic play by a director. Such actions are internal planning of images, which later should be exteriorized in a plan of external images or objects. It is a very complex cultural action.

Another case is internal action with verbal concepts. Concepts, as elements of human knowledge, are always expressed with verbal units; there is no such thing as a non-verbal concept. Gapleirn’s theory is useful because it offers the real possibility of differentiating the terms “image” and “concept,” because they are frequently mixed up in other conceptions. Concept is the interiorized generalized unit of knowledge expressed with language means. Image is the interiorized generalized unit of sensory perception expressed with representations (sounds, shapes, colors, and so on). Both verbal concepts and images of perception have to be acquired and conformed during the realization of a proper kind of cultural activity, which guarantees actions with the content of these concepts or images.

According to Galperin’s idea, these concepts and images might be developed only as results of the gradual interiorization of previous external forms of actions with external units of language and sensory images. On the level of internal action, both concepts and images are products of self-reflection and interiorization of actions and situation of usage of verbal and perceptive units. Gapleirn’s conception of a step-by-step formation of action proposes a way to understand how this interiorization is possible as a complex psychological process. According to Galperin, for psychologist it is important and interesting to study how to guide this process of formation instead of leaving it to the destiny of “spontaneous” conditions. Galperin thought that psychologists may help any sphere of cultural life in human society using this guided formation of intellectual actions. Without a doubt, the education of children, at all levels, could implement this conception. This is an important methodological explanation of the straight relation between Galperin’s theory and its application in the sphere of education (Solovieva and Quintanar, 2018). We shall consider a proposal for the gradual formation of mental actions by steps or by stages. We find no specific difference between the term “stage” or “step,” so we believe that both might be used with same meaning in relation to Gaplerin’s methodology.

## Stages of Formation of Intellectual Action

As we have expressed, the forms of existence of an action should be differentiated from the term of the stages of formation of the action. Galperin has written that the path from external plan to internal and “transformation of actions with objects into psychological phenomenon represent the way of formation of mental actions, or, in a bread sense of the word, as ideal actions” (Galperin, 1998, p. 427).

In this case we speak about the same action, which might be fulfilled on different stages or plans. So, we have to understand that only an intellectual action might be gradually formed, passing from one plan of its realization to another. Same intellectual actions, for example the counting of objects, might be represented as material, materialized, perceptual, and verbal action. The counting of objects might also be accomplished on the level of internal ideal concepts. Only an intellectual action may pass through the stages of gradual formation. Practical, physic, or other kinds of external actions always depend of only one form of realization. For instance, it is only possible to cook food in an external practical level. A drawing might be fulfilled on the level of concrete or abstract images. The writing of sentences is an external verbal action. Only intellectual action, such as solving a problem with uncertain conditions, might be fulfilled on all stages. Even more, only intellectual action may pass through all stages in a step-by-step formation.

This position is very important for teachers and psychologists, because it opens the possibility for an initial introduction of intellectual action on external plans, such as material, materialized, perceptive, or the plan of written language. The latter, as a result of interiorization, would be possible to pass to fulfilment of intellectual action on the level of internal ideal actions. So, internal actions are always intellectual actions and are a consequence of previous external actions as interiorization and reduction of external action on any of the mentioned plans (material, materialized, perceptive, and written).

The steps or the stages for gradual formation of an action are something completely different from the form or plan of action. These steps represent a methodological path for the possible formation of each intellectual action while working according to Galperin’s conception. The work might be organized at school, pre-school, a psychological service, at home practice together with children, and so on. The most common and studied way of using this conception is the organization of a formative experiment for specific concepts of education. A well-known classical example is the work of Galperin and Kabilnitskaya (1974) on the gradual formation of action of control during the action of correcting one’s own mistakes in writing. Another example is the organization of a formative experiment with artificial concepts described by Talizina (2008) and the formation of mathematical initial and advanced concepts (Talizina et al., 2017).

Our own experience is related to the organization of neuropsychological rehabilitation of adults with brain injury and of procedures of neuropsychological correction which leads to psychological development (Solovieva and Quintanar, 2015, 2018, 2019a). We are convinced that neuropsychological rehabilitation and correction is still an open and unknown field for the creative and justified use of Galperin’s theory. We are among very few authors who claim to use and cite Galperin’s theory for organization of interventional measures with neurological patients and children with learning disabilities.

According to well-known theoretical texts, the steps of the formation of intellectual actions are as follows: stage of motivation, stage of elaboration of the scheme for an orientation base of action, stage of external (material, materialized, perception, or written action), stage of verbal oral action, stage of verbal silent action, and finally, stage of internal ideal or mental action. These stages might also be called stages of assimilation or acquisition of action; this term appears frequently in books by Talizina (1984, 2018, 2019).

Table 2 shows hypothetic sequences of the stages with the content and examples of actions. Same intellectual action should be taken into account, as we try to follow the possibility of the formation of some intellectual actions from an external form to an internal form of the action. Different additional tasks (actions) should be fulfilled on each stage of the formation of intellectual action. The teacher should organize and prepare concrete attractive tasks for pupils at each stage of the formation of intellectual action. Pupils should take part in the whole process as reflexive and creative participants as we conceive learning to be a reflexive intellectual activity for pupils guided by a teacher (Galperin, 2000; Davidov, 2008; Talizina, 2019).

**TABLE 2:** Stages of formation or assimilation of a hypothetic action.

**Stage/step**	**Example of action**	**Description of content**
Motivation	Explanation of purposes and importance of the topic	Mutual analysis of correct and incorrect situations according to the topic
Elaboration of Orientation	Work with essential and necessary features of each concept/category of the topic	Elaboration of the card for orientation
External action (material, materialized, perceptive, written language)	Chosen external actions (material, materialized, perceptive, or written) useful according to the topic and previous orientation	Different tasks according to the plan of the action with the content of the topic and matter
Stage of verbal oral action	Oral explanation of the used rules/orientation	Participation in explanation and oral examples of studied situations
Stage of silent action	Work with different tasks, in which the studies’ concepts and categories might be used	Pupils have to give written examples to each other and correct mistakes in groups or independently, remembering the content of orientation
Stage of mental action	Pupils propose new creative tasks for work within categories and concepts	Work in groups or independent tasks according to the topic, possibilities for transference of knowledge

At the first stage, the stage of motivation, the teacher explains the necessity and the meaning of a concept or category, which should be started according to the matter as a specific sphere of knowledge. At the second stage, the stage of elaboration of orientation, the teacher should provide specific general orientation for the work with the concept or category. This orientation should be generalized, complete, and proper, allowing for independent work by the pupil. All features should be clear and easy to mark with external marks (symbols) understandable for all pupils.

At the third stage, the stage of external action, the most extensive work with the concept should be done. All processes are considered as a collective work between all participants. A plan of written language might be chosen. On this stage, the teacher gives examples and pupils analyze these examples. There is no individual work at this stage, all tasks are guided and done with the participation of the whole group. The tasks might be done at material, materialized, perceptive, or written plan of intellectual actions. The election of the plan of action depends on the matter, topic, and psychological age of the pupils.

At the stage of oral language, the pupils are asked to explain orally (without any other help from written language or signs) the content of the rules studied collectively in the previous stage. At the stage of silent language, pupils might be involved in different kinds of tasks, where the studies’ previous content should be used. The pupils have to remember and actualize the features of concepts or categories for these tasks.

At the last stage, or the stage of mental action, the pupils are supposed to know perfectly the previously studied content. This the stage of creative work with the possibility of using the studied knowledge in complex situations according to each discipline and topic.

Described hypothetical processes of the gradual formation or assimilation of intellectual action usually takes some time during learning and teaching. Normally, the introduction of general concepts or categories take more time at the very beginning, after which pupils get involved and start to work more rationally and show a better understanding of the problems related to the sphere of knowledge (language, mathematics, natural or social science, and so on).

After this hypothetical general explanation, we may pass to the analysis of more concrete examples of the possibility of formation or assimilation of intellectual action.

## Example of Formation or Assimilation of Intellectual Action

We have chosen the same action for all stages, so that the reader might be convinced of the process of formation of the same intellectual action by stages instead of putting different examples of different stages at all stages. The action, chosen for Table 2, is the action of the classification of grammar categories. The whole procedure with this action is provided in one of the books by the authors (Solovieva, 2016).

The work with grammar rules might be organized as productive and creative tasks, interesting and attractive for students. The work is important at any stage and with any groups of participants, where it is necessary to introduce grammar categories for the first time. Our previous experience with the work was successful with second-grade students of a primary school in Mexico, which was published in a different article (Solovieva et al., 2019). The goal of this article is to argue for the advantages and possibilities of usage of the conception of the gradual formation of intellectual action according to Gaplerin and Talizina, but not to expose any kind of specific method. So, we ask the reader to take into account that the content of Table 2 is just an example and not the whole description of any method. For more details of work with the introduction of written language at school, we ask the readers to look for other publications (Solovieva, 2015, 2016; Rosas and Solovieva, 2017, 2018; Solovieva et al., 2018; Talizina, 2019).

At the first stage, the stage of motivation, the teacher explains that the words form different categories and that it is useful and interesting to study in order to understand the structure of our oral and written speech. With clear understanding of this structure it would be easier to study other languages, to read books, and to produce independent texts.

At the second stage, the stage of elaboration of orientation, the teacher explains that the words might be classified in specific types: word grammar categories. Each category has proper features and may differ by sense (what does the word mean), the way of behavior (how can the word change), and the structure (which parts of the word change and which don’t change). The teacher may propose a creative way of putting down these three features with specific signs. Pupils can take part and propose the signs for marking the sense, behavior, and structural parts of words.

At the third stage, the stage of external action, the plan of written language might be chosen. During this stage, the teacher would give examples of the words written on the blackboard or in notebooks and the pupil would use the signs, proposed at the stage of orientation, to mark and discuss the meaning, changeable or static behavior, and the constant and changeable parts of each word. Pupils may underline examples and variants of words with different colors or marks, according to previously established agreements.

At the stage of oral language, children are asked to explain orally (without any other help of written language or signs) the content of the rules for general grammar categories.

At the stage of silent language, pupils might be involved in different kinds of tasks, where the studies’ previous content should be used. The pupils have to remember and actualize the features of the grammar categories for these tasks. An example of such a task might be the correction of mistakes in notebooks or in texts.

At the last stage, or the stage of mental action, the pupils are supposed to know perfectly the previously studied content. This is the stage of creative work with the possibility of passing on the studied knowledge to complex situations. An example might be the explanation of grammar categories in a foreign language. Other proposals of complex creative tasks are, obviously, possible.

The whole process may be achieved in one week for the introduction of general grammar categories. Afterward, the teacher may start work with concrete grammar categories such as substantive, adjective, verb, adverb, article, and so on.

Table 3 demonstrates that the stages of formation of action might be provided by, guided, and designed by previous orientation. The stages would be organized and fulfilled in the context of a school learning activity. Such an activity is always a kind of joint work with other pupils and a teacher, who is the provider of the content of orientation base of action.

**TABLE 3:** Stages of formation or assimilation of the action of identification of general grammar categories.

**Stage/step**	**Example of action**	**Description of content**
Motivation	Expression of needs and possibilities of study of one’s own language	Mutual analysis of correct and incorrect verbal expressions, comparison of sentences and texts
Elaboration of Orientation	Work with essential features of grammar categories such as: – Semantic features – Grammar features – Morphological features	Elaboration of the card for general introduction of the concept of grammar features: – What does the word refer to? – What changes might be done with the word? – What is the structure of the word?
External action (material, materialized, perceptive, written language)	Written action and symbolic materialization: – identification different of sense of the word – changeable grammar elements – structure of constant elements and changeable elements of words	Written tasks of pupils with identification of each element in different words: sense, grammar changes, morphological elements with are constant and which change
Stage of verbal oral action	Pupil have to explain the used rules	Pupils have to give oral examples to each other
Stage of silent action	Pupils have to think about examples with and without mistakes for the identification of grammar categories	Pupils have to give written examples to each other and correct mistakes
Stage of mental action	Pupils propose new creative tasks for work with the categories	Work with grammar categories in other languages

The topic of orientation base of action is closely related to the conception of the formation of actions by stages. It is even possible to say that, without the concept of orientation base of actions, the whole conception would lose its sense. Orientation base of action is quite a complex concept, which includes the formation of internal images of psychological action. Actually, the formation of action by stages, on the basis of an orientation base of action, conducts the formation of internal images of this action. In other words, this enables the visualization of images as a result of actions with the objects on the basis of orientation base of action. The realization of actions with objects enables the formation of internal images of situations, features, objects, and actions. Galperin speaks about different kinds of mental images, which might be formed as a result of actions with objects.

## Discussion and Conclusions

Our main conclusions refer to the necessity of constant reflection and actualization of the main concepts of activity theory as logical and consistent continuations of the basic positions of Vigotsky’s historical and cultural paradigm of development. The possible lines of such consideration are as follows. Vigotsky established the process of development as the main object of psychological study. Activity theory with methodology of the formative experiment allows to reaffirm Vigotsky’s proposal (Talizina et al., 2010). In this case, development might be studied not only spontaneously but also as a guided and organized process with the inclusion of the consideration of specific stages and features. Galperin’s conception of stages for the gradual formation of actions permits the operationalization and creation of formative experiments. A comparison of results of the introduction of initial verbal concepts and learning of written speech at a primary school based on this conception with results of traditional methods of teaching show advantages of Galperin’s conception adapted to conditions of the Spanish language in Mexico (Torrado et al., 2018). A creative approach needs to be designed and approved, and that would be a most important contribution the historical-cultural approach and activity theory.

The actual moment of development of this approach requires new examples of formative experiments and the profoundization of concepts and methodology. The conception of the gradual formation of actions or interiorization requires integration with concrete psychological studies. The field of application of such formative experiments may be broad and should be applied to both normal and special education. Specific stages and forms of actions might be created and approved for practical, artistic, and intellectual actions. A methodological strength of the conception of the gradual formation of psychological actions is still to be discovered.

The authors of the article were interested not only in understanding the conception of the gradual formation of actions by stages and to study examples of the formation proposed by Galperin and his followers, but also to design proper formative experiments based on this conception. Such experiments took place over ten years in an experimental college for pre-school and school children organized by the authors in the City of Puebla (Mexico)^1^. The authors are convinced that future experimental works and theoretical considerations are necessary in different social and cultural contexts, health conditions, and levels of education with broad participation from teachers, educators, psychologists, and neuropsychologists in order to design and approve new methods for teaching and correction which lead to psychological development of the pupils. Galperin’s methodology deserves major understanding, acceptance, and reflection from researchers and specialists.

## Data Availability Statement

The original contributions presented in the study are included in the article/supplementary material, further inquiries can be directed to the corresponding author.

## Author Contributions

All authors listed have made a substantial, direct and intellectual contribution to the work, and approved it for publication.

## Conflict of Interest

The authors declare that the research was conducted in the absence of any commercial or financial relationships that could be construed as a potential conflict of interest.

## Footnotes

1 www.colegiokepler.edu.mx
